# Unraveling Elusive Boundaries: A Comprehensive Framework for Assessing Local Food Consumption Patterns in Nova Scotia, Canada

**DOI:** 10.3390/foods12183492

**Published:** 2023-09-20

**Authors:** Sylvain Charlebois, Marie Le Bouthillier, Janet Music, Janèle Vézeau

**Affiliations:** 1 Agri-Food Analytics Lab, Dalhousie University, Halifax, NS B3H 4R2, Canada; 2 Centre NUTRISS—Nutrition, Santé et Société, Institut sur la Nutrition et les Aliments Fonctionnels (INAF), Université Laval, Québec, QC G1V 0A6, Canada; marie.le-bouthillier.1@ulaval.ca; 3 Department of Sociology and Social Anthropology, Faculty of Arts and Social Science, Dalhousie University, Halifax, NS B3H 4R2, Canada; jlmusic@dal.ca; 4 Canadian Agri-Food Foresight Institute, Halifax, NS B3H 4R2, Canada; janele.vezeau@cafi-icpa.ca

**Keywords:** local food, consumer perceptions, experimental methodology, Canada

## Abstract

Promoting local food consumption for economic growth is a priority; however, defining “local” remains challenging. In Nova Scotia, Canada, this pioneering research establishes a comprehensive framework for assessing local food consumption. Employing three data collection methods, our study reveals that, on average, Nova Scotians allocate 31.2% of their food expenditures to locally sourced products, excluding restaurant and take-out spending, as per the provincial guidelines. The participants estimated that, in the previous year, 37.6% of their spending was on local food; this figure was derived from the most effective method among the three. However, the figure was potentially influenced by participant perspective and was prone to overestimation. To enhance accuracy, we propose methodological enhancements. Despite the limitations, the 31.2% baseline offers a substantial foundation for understanding local food patterns in Nova Scotia. It serves as a replicable benchmark for future investigations and guides researchers with similar objectives, thereby establishing a robust research platform.

## 1. Introduction

Local food consumption holds the potential to invigorate regional economies, keeping wealth within communities while bolstering the livelihoods of local farmers [1,2,3]. Consumers can exhibit a notable propensity to spend on locally sourced agricultural products [4,5]. Hence, as part of regional economic strategy, governments should emphasize raising the proportion of local food spending relative to overall food spending.

However, amidst the fervor for local food, a critical challenge has arisen: to define precisely what constitutes “local”; this has proven to be a perplexing endeavor. While there is a consensus that local food originates within a confined geographical sphere, the consensus eludes us when it comes to quantifying the limits, whether in terms of kilometers or regions [6,7,8]. Additionally, consumer expectations introduce further complexity, with demands for attributes such as small-scale, artisanal production methods [5,9]. Establishing a definitive definition for local food is thus an essential initial step in fostering community growth through its promotion.

Subsequently, assessing the baseline of local food consumption, namely the proportion of the total food budget allocated to local products, emerges as the second pivotal phase in expanding its reach. Typically, evaluating local food consumption hinges on the examination of individual behavioral and attitudinal traits. For instance, intentions to purchase local food are often gauged through measurements of factors like attitudes toward local food consumption and the subjective norms influencing such decisions [10,11]. In some cases, researchers resort to indirect metrics, such as crop receipts or farmer production data, to estimate local food expenditure, as exemplified by a study in Nova Scotia [10,11], which calculated that in 2008, 13% of the food budget directly benefited local farmers [8]. Nonetheless, more direct methodologies are warranted for a precise analysis of actual local food consumption, especially with regard to its share within the overall food budget [12,13,14].

However, when utilizing surveys prompting participants to recall local food purchases in dollars or as a percentage of total expenditure over a specific period, biases come into play. Notably, the desirability bias is manifested, where individuals tend to over-report socially desirable behaviors while under-reporting those deemed less acceptable [7,15,16]. While this phenomenon is well documented in the study of sustainable food practices, adjusting for it within the context of local food remains a challenge [1,4]. Therefore, it is assumed that people tend to over-report their local food consumption due to its societal desirability, though the extent of this over-reporting remains uncertain.

Additionally, the imperfect recall bias influences results, as individuals tend to be better at recalling information that serves their interests than providing an accurate account of past events [17,18]. In particular, given the prevalence of credit card use for food purchases, individuals may underestimate their spending; however, the quantification of the scale of this under-reporting remains elusive [19,20,21].

To compound matters, studies involving food consumption often reveal misreporting when participants are asked to assess nutrient intake, with errors of omission (forgetting to mention certain items) or errors of commission (reporting items outside the specified timeframe) [8,22,23,24]. The most effective strategy to mitigate such biases involves employing a diverse array of methods to measure food consumption [25,26,27]. Triangulation, the simultaneous use of multiple methods to observe a phenomenon, substantially enhances the credibility and validity of findings [28,29]. It is important to note that under-reporting tends to be more common when recalling food intake, especially among individuals with higher body mass indexes, but over-reporting is not uncommon either [30].

Considering the scarcity of previous research in this domain, the present study undertakes a novel approach to addressing the significant gap in the understanding of local food consumption within economic contexts. Prior to this investigation, no similar studies were documented, rendering this study’s undertaking a pioneering effort. The central hypothesis posits that by formulating a comprehensive methodology to quantify local food consumption as a proportion of total food expenditures, a more accurate assessment can be achieved and thereby contribute to the enhancement of local economies. However, this study encounters a notable challenge owing to the absence of previous research; thus, the development of a fresh analytical framework from the ground up is required.

The primary aim of this study is to introduce an innovative methodology for the precise estimation of local food consumption within economic parameters. The significance of this objective stems from its potential impact on the growth of local economies. Accurate quantification of the share of total food budgets allocated to local food consumption is pivotal for effective forecasting and strategic interventions aimed at encouraging consumer behavior changes [31]. Given the absence of any previous work in this specific area, this study seeks to pioneer a foundational understanding of local food consumption in economic terms.

To achieve this study’s objective, a multifaceted approach was devised. Initially, a comprehensive definition of “local” was formulated, encompassing 15 distinct food categories that collectively span the breadth of the food spectrum. Subsequently, an online questionnaire was administered to consumers, employing three distinct methods to gauge the distribution of their budgets between local and total food expenses. This methodological triangulation was employed to mitigate potential biases in the data collection. To ensure precision, one outcome was adjusted to account for food spending variations, establishing it as the most reliable approach. Method 2 emerged as the most suitable and dependable method; thus, it formed the bedrock of the tentative methodology adopted in this study.

The methodology employed in this study leverages the robustness of Method 2, which emerged as the most accurate approach through rigorous analysis. This pioneering methodological framework addresses the dearth of prior research in the field, forming a foundational step toward comprehending local food consumption within economic contexts. The ensuing results and discussions elucidate the insights garnered through this innovative approach, offering fresh perspectives and potential avenues for future exploration. As the first of its kind, this study lays the groundwork for subsequent research endeavors in this uncharted territory, promising a deeper understanding of the economic implications of local food consumption.

## 2. Materials and Methods

### 2.1. Survey Design

A quantitative survey in English was designed and administered online by Angus Reid on the platform Qualtrics and was available for 13 days (7–20 April 2023). The final questionnaire consisted of 62 questions, with many identical questions that varied only in terms of the food category considered. First, general questions about food spending were asked, followed by questions regarding specific food categories. Next, questions about the participants’ local food consumption were posed, followed by questions regarding demographics. The average time to complete the survey was 51 min.

The developmental process for our research instrument was significantly influenced by extensive literature reviews, with a particular focus on key reports published in recent years on food consumption trends and methodologies [32,33,34]. To enhance the instrument’s credibility and validity, a meticulous pretesting and piloting phase was undertaken to ensure that the questions effectively captured the participants’ local food consumption behaviors and preferences.

Prior to administering the instrument to the study participants, we took great care in clarifying the definitions of “local food” to ensure a common understanding among the respondents. This step was vital to eliminate any potential ambiguity or misinterpretation in the participants’ responses.

To estimate the amount spent on local food, our study employed three distinct methods; this was a deliberate strategy aimed at triangulating the results and mitigating potential biases. Method 1 involved direct inquiries regarding the participants’ total food expenditures over the previous 12 months, followed by specific queries about their spending on local food during the same period, all in dollar amounts. The research team then translated these figures into percentages for analysis.

Method 2 introduced a more detailed approach, prompting the participants to estimate their expenditures on specific local food items within defined categories over the previous year. These estimates were then weighted based on overall food spending and subsequently averaged to derive the total local food consumption as a percentage of the total food budget.

Method 3 directly solicited the participants’ perceptions of their local food spending over the previous 12 months; this was expressed as a percentage. All the questions related to financial estimates employed a continuous rating scale, requiring the participants to position a cursor to indicate their responses accurately.

Additionally, our instrument encompassed a range of supplementary questions designed to provide a comprehensive understanding of local food spending behaviors. These questions covered topics such as the timing of the participants’ highest local food purchases, the reasons influencing their decisions to buy locally or not, and the specific local products they purchased most frequently. Furthermore, the participants were asked to identify the criteria important to them when purchasing organic products and to indicate their preferred sources for obtaining these products. These inquiries yielded valuable supplementary insights into the nuances of local food consumption, contributing to a holistic and comprehensive analysis.

This study was conducted with regard to Nova Scotia’s local food system and economic development. This study was conducted in collaboration with Perennia Food and Agriculture, a Crown corporation with the mission to support growth, transformation, and economic development in Nova Scotia’s food and agricultural sectors. As per their request, the present research team had to develop a methodology to assess consumption of local food in Nova Scotia to allow the projection and forecasting for economic development.

### 2.2. Local Food Definitions (15 Food Categories)

An initial version of local food definitions was provided by Perennia Food and Agriculture to the research team. These definitions had been developed and approved by the Department of Agriculture. The research teams revised these definitions, and the two teams agreed on a final version that was consistent with Nova Scotia’s economic plan but was as simple as possible for consumers and covered most of the food spectrum. All the questions mentioned that the food consumption excluded restaurants or take-out meals. See Appendix A for these 15 definitions.

### 2.3. Data Collection

A total of 511 participants were recruited by Angus Reid through its online database of eligible participants; these participants completed the entire survey. The demographic composition of the participants was diverse, with ages ranging from 18 to 90 and older, reflecting a broad spectrum of Nova Scotia’s population. In terms of gender distribution, the sample included a balanced representation of both male and female participants. Economic status within the sample encompassed a range of income brackets, with participants from various socioeconomic backgrounds, and thus captured the economic diversity within Nova Scotia’s populace [35,36].

The selection of this sample size is robust and defensible in the context of Nova Scotia’s market for several compelling reasons. Given Nova Scotia’s relatively modest population size, which stands at less than 1 million people, the sample size of 511 participants effectively represents a diverse cross-section of Nova Scotia’s demographic and socioeconomic landscape, ensuring comprehensive coverage of the various factors influencing local food consumption [35,36]. This inclusivity enhances this study’s external validity, permitting the extraction of meaningful insights that can be generalized to the broader Nova Scotia population.

Moreover, the sample size falls well within the range necessary to achieve statistical significance, safeguarding against coincidental findings and substantiating the likelihood that this study’s results genuinely reflect prevalent trends and patterns in local food consumption behavior within Nova Scotia [37,38]. The geographic and cultural coherence of Nova Scotia as a relatively compact region further reinforces the suitability of this sample size as it facilitates a more concentrated focus on local dynamics and nuances within the local food market [37,38].

Finally, this study’s deliberate selection of the individuals primarily responsible for food purchases refines the scope of inquiry, making the sample size of 511 participants especially appropriate for delving into the intricacies of this specific facet of local food consumption behavior [39,40].

### 2.4. Data Analysis

Data were compiled on the platform Qualtrics and then extracted by the research team.

The selection of three distinct methods for assessing local food consumption aimed to provide a comprehensive and robust understanding of this complex phenomenon. Each method was deliberately designed to address potential biases and variations in the data reporting. Method 1 employed a straightforward approach, converting total expenditures into percentages of local food spending, allowing a basic overview of local food consumption. In contrast, Method 2, conducted through R Notebook, introduced a more intricate calculation, factoring in mean and median responses while considering the unique characteristics of different food categories. This method sought to mitigate potential over-reporting biases. Moreover, it incorporated weighted percentages based on Statistics Canada’s 2019 food expenditure data for Nova Scotia, enhancing the accuracy of the results. Notably, Method 2 carefully handled cases where the respondents reported zero dollars spent on local products. Finally, Method 3 utilized Qualtrics to yield a mean response automatically, providing a baseline for comparison. In the data analysis phase of our study, we employed a structured approach designed to ensure accuracy and reliability in estimating local food consumption percentages. We conducted three distinct methods of analysis, each tailored towards addressing potential biases and variations in the data reporting.

For Method 1, we initially calculated the mean responses for the total amount spent on all food and the total amount spent on local food. These figures were then transformed into percentages representing the proportion of the total food budget allocated to local food. This method involved straightforward mathematical calculations to obtain local food expenditure percentages.

Method 2 necessitated a more intricate analysis process; we utilized R Notebook as our chosen statistical software. Data from the CSV file, exported from the Qualtrics platform, were processed using R. We performed calculations to derive percentages for specific food categories, such as “Meat” and “Dairy + Eggs”. This involved dividing reported expenditures on local products by the corresponding percentage representation for each category. Subsequently, we combined the dollar values for both the local and the total amounts within these categories and computed the local food expenditure percentages. It is essential to note that, due to the nature of survey responses, when participants indicated zero dollars spent on a local product, we faced uncertainty regarding whether this was due to a lack of purchase or simply not purchasing locally. To address this, we applied specific assumptions, setting total dollars for the component to zero in cases of zero local dollars spent. Similarly, if a response included local dollars but had a local percentage of zero or was non-responsive, we assumed the entire expenditure was local (i.e., 100% local). Statistics for individual categories were calculated without weighting, and missing responses (excluding those set to zero) were omitted.

To further refine the analysis, we introduced weighted percentages for each food category, utilizing data derived from the 2019 Statistics Canada food expenditure figures specific to Nova Scotia. We assigned specific weights to categories such as “Honey” and “Maple Syrup” due to their unique characteristics and the potential for overestimation. These weighted percentages were then multiplied by the respondents’ local spending percentages, yielding an adjusted local food expenditure percentage. Any values exceeding 100% were discarded to maintain data integrity.

As poultry and dairy products in Nova Scotia are supply-managed, we made a deliberate choice to omit these categories from the results, which required a re-weighting of the total. In this context, the “Dairy” category was entirely excluded, while the “Meat” category was considered to exclusively comprise red meat, with an equal weighting applied.

For Method 3, the response was automatically generated by the Qualtrics platform and represented the mean response from the participants.

Ultimately, after meticulously considering the outcomes of all three methods, Method 2 was selected as the most accurate. This choice was based on the recognition that several biases within the data likely led to over-reporting, and Method 2 yielded the lowest percentage, providing a more conservative estimate of local food consumption. The results from the remaining questions were compiled within the Qualtrics platform and subsequently extracted by the research team for comprehensive analysis. This multifaceted approach to data analysis ensured that our study’s findings accurately reflected the complexities of local food consumption behavior in Nova Scotia while minimizing potential biases.

## 3. Results

### Characteristics of the Survey Participants

The total sample consisted of 511 participants from Nova Scotia, aged 18 to 90 years and older, with a similar distribution among men (41.7%) and women (55.1%). Most of the survey respondents lived in small towns or rural areas (52.8%), with the rest distributed between the urban core (18.2%) and suburban areas (28.2%). Most lived in a house/apartment with their family/spouse (70.5%). Most were married (69.1%), with at least one child (64.8%) and a college degree or higher (74%), and had made at least CAD 50,000 in their last year of employment (60.5%). Most of the participants had no dietary preferences (78.3%). Most of the participants bought groceries for themselves and one other person (47.4%).

**Figure 1:**
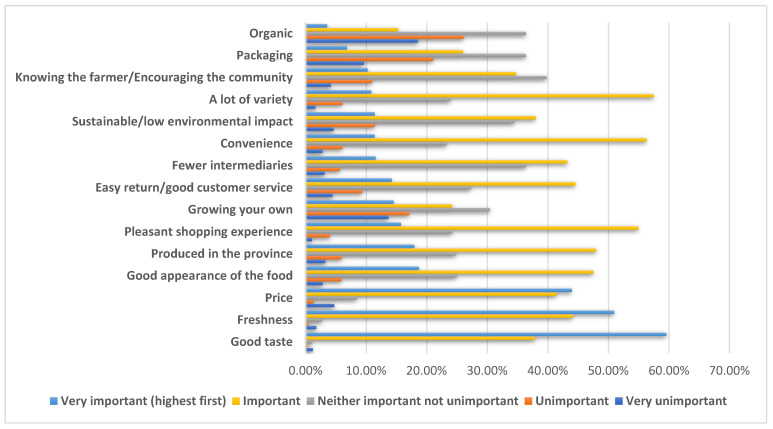
Attributes and importance when buying food products.

When asked to rate the level of importance of certain attributes when purchasing food, our participants indicated that good taste, freshness, and price were the most important. The least important criteria were knowing the farmer/encouraging the community, packaging, being organic, and growing your own. Figure 1 shows the distribution of the results by answer choice (from very unimportant to very important); the results are ranked according to which attribute obtained the highest score for the very important answer choice. Although growing your own did not rank last in terms of highest ranking for the very important response choice, the mean of the responses for this attribute scored second for the very unimportant response choice.

In terms of the outlets where they typically purchase produce, our participants indicated that the local supermarket, the major supermarkets, and the local specialty stores (butcher shop, bakery, fruit and vegetable store) were the places where they most often purchased their food. As for producing it, their own backyard and community gardens scored among the top five outlets. The least-used outlets were farmers selling produce directly, community-supported agriculture (CSA), and individual online producers. Table 1 shows the distribution of results according to the response choice (“never” to “always”), ranked by the outlet that scored the most points for the “always” response choice.

Table 2 shows the results of the participants’ responses to the question on how much they spent on food in total over the previous 12 months and how much they spent on local food over the same period. The average for total food per month was CAD 681.1, while the average for local food per month was CAD 317.8. The participants had seen the definition of the local food item before they estimated their total consumption, and they were asked to recall their purchases over the previous 12 months.

We used these averages in the first method to calculate food spending (Method 1): the average of the local food spending and the average of the total food spending in dollars; then, we translated these into percentages. When calculated with these numbers, 46.7% of the total money spent on food per month was spent on local food, as averaged over the previous 12 months.

**Figure 2:**
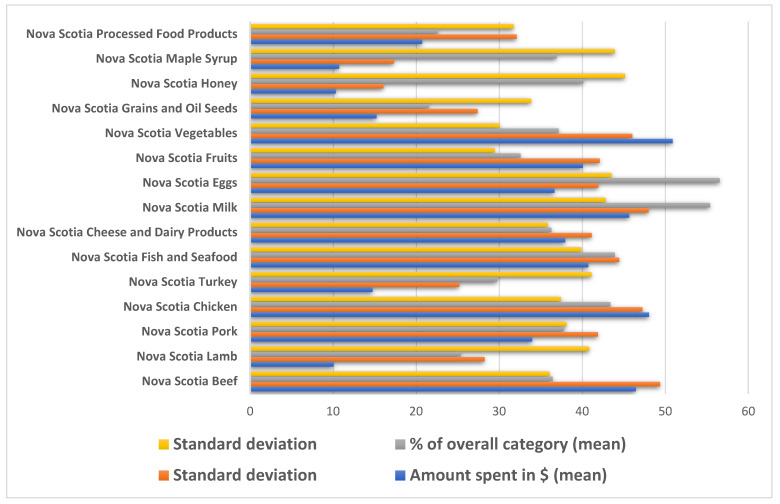
Summary of amount spent and percentage of total spending on local food.

In Figure 2, we can see the participants’ responses when asked about the dollar value spent on local food over the previous 12 months, according to our definitions in Appendix A. We can also see the estimated percentage of the overall category. Notably, local beef, chicken, milk, and vegetables were the items on which the most money was spent, while the amount spent on local milk and eggs accounted for the largest share of the budget for this food category. Processed products and grains and oil seeds were the least locally bought categories. The participants always saw the definition of the local food just before they estimated it, and they were asked to recall their purchases over the previous 12 months.

Table 3 shows the spending percentages by Nova Scotians in 2019, from Statistics Canada [15]. These numbers have been converted to percentages.

Table 4 shows the results of Table 5 for local food consumption, with the Statistics Canada weight categories.

Table 5 shows the results when the respondents were asked how much they had spent on local food over the previous 12 months. The mean was 34.8%. This number was used as the average for Method 3 to estimate local food spending. The participants had seen the definitions for each local food item before they estimated their total consumption and were asked to recall their purchases over the previous 12 months.

Table 5 displays a summary of averages for each method. Method 1 is a calculation of the mean between the reported total spending on food and what was spent on local food in dollars; this was translated into percentages. Method 2 is an average of all the food categories, weighted for sales, in percentages. Method 3 is the self-reported data when we asked the participants directly; these data were translated into percentages. Then, the average of the three methods was presented.

## 4. Discussion

### 4.1. Method 2

We investigated the assessment of local food consumption within Nova Scotia from the perspective of the consumers. This inquiry employed a tripartite approach in an online survey, encompassing self-disclosure of monetary disbursements, self-disclosure of expenditures across 15 delineated food categories, and direct self-disclosure in percentages. Among these methods, Method 2 emerged as the most meticulously accurate, furnishing an estimation of 31.2% (excluding commodities under supply management). This preference for Method 2 is rooted in its propensity to generate the most conservative estimate vis à vis the other methodologies, which probably approximates veracity due to the likelihood of various biases inducing an overstatement of outlays by participants.

The collective mean derived from these three methodologies provided a coarse mean value: the respondents determined that 37.6% of their outlay for alimentary items over the preceding 12 months was attributed to local victuals. However, this final estimation likely inclines toward an embellishment owing to the diverse biases inherent in the survey. Notably, nearly half of the participants reported procurement for personal consumption as well as for one additional individual, intimating that this percentage would be diminished should we recalibrate it per inhabitant rather than per primary purchaser.

### 4.2. Methodological Challenges

It is imperative to underscore that our findings predominantly portray the consumer’s perspective as opposed to reflecting actual consumption patterns. Given this premise, coupled with inherent sources of bias such as desirability bias, fallible recollection, and inaccuracies in food reporting, the resultant percentage projection is inclined toward overvaluation relative to authentic consumption. Furthermore, an appraisal of the ratio between Method 1 and Method 2 (denoting declared monthly expenditures vis à vis declared category-specific expenditures) revealed inconsistency among the participants. Specifically, 70.4% overestimated their local consumption based on monthly monetary records, a mere 1.3% remained congruent, and 28.3% underestimated their local consumption based on their recorded monthly expenditures. This disjuncture in participant computations underscores the exigencies involved in triangulating the results, given the discernible variations across methodologies. It also underscores the conjecture that the recall of monetary disbursements may yield an overestimate, while direct percentage recall could yield an understated projection.

In summary, the yielded figure predominantly encapsulates the consumer’s perception, illuminating their conception of local alimentary acquisitions rather than an accurate manifestation of real-world purchases. Notwithstanding this limitation, the proposed methodology exhibits a high degree of replicability, facilitating the monitoring of consumption trends over a period of time.

### 4.3. Data Deficit

To enhance the precision of this study, a potential avenue involves simplifying the taxonomy of local victuals. Streamlining the definitions could potentially ameliorate outcomes and confer a more faithful representation of actual consumption patterns. This proposition is bolstered by the participants’ candid feedback in response to an open-ended inquiry, wherein numerous participants expressed difficulty in recollecting purchases over the previous year and in matching them with the complex descriptions provided for the local foods [35]. This emphasis on the challenge of recall underscores the potential efficacy of simplifying local food delineations. Employing unambiguous definitions across all food categories could notably enhance consumer comprehension and memory retrieval. Moreover, anchoring these definitions in production and distribution metrics could offer further advantages. Given the social cachet associated with purchasing local produce, a climate of definitional ambiguity might predispose individuals to opt for overestimation in cases of uncertainty rather than underestimation.

Incorporating retailer data as a yardstick for local consumption could conceivably augment the precision of our findings. The intrinsic interplay between local food consumption, production, and the broader alimentary ecosystem renders isolated quantification challenging [41,42,43]. Integrating data from retailers, particularly local food sales metrics, holds the potential to furnish valuable insights. However, the acquisition of such data is hindered by conceivable confidentiality constraints and the incongruence between the retailers’ classification of local food and the taxonomies employed in this study. Resolutions might involve alignment with retailer definitions, long-term partnerships to facilitate data sharing, and the possible revaluation of local food categories. Such measures could supplement our extant findings with a confirmatory layer of validation.

An additional limitation pertains to the relatively modest sample size in relation to Nova Scotia’s populace. Nevertheless, the attendant margin of error remains within acceptable limits, reaching 4.4% in 19 cases out of 20 (at a 95% confidence interval). This deviation aligns with the customary tolerance range of 4% to 8% within a 95% confidence interval. Lastly, while our local food definitions were conceived with comprehensiveness in mind, it is conceivable that certain alimentary items were inadvertently omitted, potentially engendering distortions. Harmonizing with Statistics Canada’s food categories could potentially attenuate this drawback and bolster this study’s construct.

Within the context of Nova Scotia, the inclusion of dairy, poultry (chicken and turkey), and eggs in supply management programs engenders an artificial elevation in these categories, encompassing production, production costs, and imports, to confer stability to farmers and consumers in their exchange [44]. This regulatory framework affords local producers a favorable milieu for sales and profit generation, with mitigated competition from imports. Consequently, our study reflects an embellished valuation for these categories relative to their characteristic absence in supply management programs. Upon the exclusion of these categories, the Method 2 computations yielded an aggregate of 31.2% for all other categories not covered by supply management programs. This deduction suggests that, beyond supply-managed foods, the participants’ expenditure equates to 31.2% of their income being allocated to local victuals. This observation assumes added significance in light of the mutable nature of these management programs, which remain susceptible to negotiations and political vicissitudes, necessitating a mindful perspective on potential programmatic changes.

### 4.4. Importance of Study

Insights garnered from supplementary survey queries underscore avenues for local farmers to bolster sales. The participants prioritized attributes such as flavour, freshness, and price in accordance with analogous research findings [45,46]. This congruence offers an opportunity for local farmers, given the heightened demand during the summer months (July, August, and June) when a profusion of farmers’ markets facilitates greater access to diverse produce, especially fruits and vegetables. This confluence engenders an auspicious context in which farmers can capitalize on their unique selling points, facilitating the sale of exceptionally fresh commodities at competitive prices while circumventing intermediaries. Capitalizing on these attributes during this temporal window could confer a competitive edge, resonating with consumers’ preferences and demarcating local produce within a saturated marketplace.

Furthermore, our survey findings underscore the need for an enhancement in consumer-oriented labeling strategies. Evidently, a noteworthy impediment to the procurement of local foods lies in their limited availability across diverse categories. While this scarcity might partially stem from the inherent seasonality of certain commodities, it is conceivable that the absence of unambiguous definitions and distinct labeling mechanisms contributes substantively to this predicament. As previously alluded to, the implementation of more lucid taxonomies has the potential not only to facilitate consumers’ memory retrieval processes but also to catalyze more perspicuous labeling practices on the part of retailers. This assertion aligns cohesively with the observation that consumers exhibit a pronounced willingness to engender economic sustenance within their local milieu—an imperative that emerges as a primary driver behind the decision to patronize local markets. Notably, there is a discernible tendency among individuals to procure items such as eggs, seafood, chicken, milk, and beef from local sources. This conspicuous inclination proffers a distinctive opening for retailers and agriculturists to channel their efforts toward these specific categories, thereby accentuating the unique attributes of local products. Realizing this potential entails the implementation of clearer labeling conventions, thereby enhancing accessibility and visibility and subsequently amplifying the prospects of consumer engagement and adherence to local sustenance paradigms.

### 4.5. Environmental, Managerial, and Policy Implications

#### 4.5.1. Direct Implications for Nova Scotia’s Farmers, Businesses, and Consumers

The discussions and findings presented in this paper hold tangible implications that resonate directly with Nova Scotia’s farmers, businesses, and consumers, bridging the gap between theoretical study and real-world applications.

#### 4.5.2. Sustainable Economic Boost for Farmers and Local Businesses

This study’s revelations concerning consumer preferences for specific local food categories like eggs, seafood, chicken, milk, and beef provide a roadmap for local farmers and businesses to strategically align their efforts. By capitalizing on these consumer preferences and adopting transparent labeling practices, farmers can cater directly to market demands. This alignment enhances their visibility and positions them as champions of local, sustainably produced goods, creating a competitive edge within the market.

#### 4.5.3. Strengthening Local Food Networks

The insights garnered from this study can facilitate collaboration between farmers, local retailers, and consumers. By focusing on the preferred categories highlighted by consumers, businesses can create mutually beneficial relationships within local food networks. This can involve fostering partnerships with community-supported agriculture (CSA) initiatives, local farmers’ markets, and supply chains, thereby cultivating a thriving local food ecosystem that bolsters economic sustenance within the province.

#### 4.5.4. Enhancing Consumer Awareness and Education

The findings underscore the need for well-designed marketing campaigns and targeted consumer education initiatives. These efforts can enlighten consumers about the tangible benefits of opting for locally produced foods. By dispelling misconceptions and conveying a clearer understanding of local food categories, businesses can foster a deeper connection between consumers and the local food ecosystem, driving increased patronage and loyalty.

#### 4.5.5. Positive Environmental Impact and Sustainable Practices

This study’s alignment with principles of sustainable consumption and production aligns well with Nova Scotia’s commitment to environmental stewardship. The consumers’ willingness to support the local economy and consume regionally produced goods presents an opportunity to minimize the ecological footprint associated with long-distance transportation. Farmers can respond by adopting sustainable agricultural practices that align with these values, further strengthening the connection between local food and environmental conservation.

#### 4.5.6. Informed Decision Making and Policy Formulation

This study’s insights can inform the decision-making processes of policymakers in Nova Scotia. Clear and standardized labeling regulations for local foods, as suggested by this study, can enhance transparency for consumers. Additionally, policymakers can consider crafting incentives, subsidies, or technical support to enable farmers to extend growing seasons, addressing the challenge of seasonality and bolstering the availability of local produce year-round.

#### 4.5.7. Addressing Market Discrepancies and Supply Chain Management

This study’s identification of discrepancies between self-reported expenditures and direct percentage reporting underscores the importance of robust supply chain management and accurate record keeping for retailers and producers. Businesses should consider aligning their reporting mechanisms closely with consumer behavior to ensure accurate assessments, which can influence pricing, stock management, and overall business strategies.

In summary, this study’s findings traverse the academic realm and directly intersect with Nova Scotia’s economic, environmental, and policy landscapes. By translating these findings into targeted actions, farmers, businesses, and policymakers can collaboratively contribute to a more vibrant, sustainable, and locally focused food system that benefits the province’s inhabitants and its environment alike.

Policymakers in the province could consider introducing or refining regulations that mandate clear and standardized labeling for local foods. Such regulations could enhance transparency, reduce consumer confusion, and enable more informed decision making. To address the challenges posed by the seasonality of local products in the province, policy interventions might include incentives, subsidies, or technical support for farmers to adopt practices that extend growing seasons and enhance the availability of local produce year-round.

Collaborative efforts between researchers, policymakers, and retailers to improve data collection methodologies could lead to more accurate assessments of local food consumption. This could involve exploring partnerships with retailers to access sales data and align data categories with research parameters.

Policymakers could bolster local food networks by facilitating collaborations between farmers, retailers, and consumers. This could entail supporting farmers’ markets, community-supported agriculture (CSA) initiatives, and local supply chains, thus nurturing a robust local food ecosystem.

The implications derived from this study offer a comprehensive framework for environmentally conscious decision making, strategic management, and informed policy development for a province like Nova Scotia. By leveraging the insights gained from consumer preferences, challenges, and opportunities related to local food consumption, stakeholders can collectively contribute to a more sustainable, resilient, and thriving food system.

## 5. Conclusions

Our study’s participants estimated that approximately 31.2% of their food expenditures over the previous 12 months were allocated to local foods, excluding restaurants, take-out meals, and supply-managed categories (34.2% when including supply-managed categories). While insightful, it is prudent to adjust this consumer perspective to better align with actual consumption, considering the fact that a slight overestimation likely exists. This baseline, derived from Method 2, offers a valuable initial assessment of consumption, and the method’s accuracy and reproducibility make it particularly valuable.

Triangulating methods to mitigate bias underscored Method 2’s efficacy in approximating reality. This pioneering analysis sets the stage for future studies with similar aims. Opportunities for enhanced accuracy are explored, such as leveraging retailer data through agreements. Clear communication emerges as crucial, whether through improved definitions or the labeling of local products.

The future of local food consumption in Nova Scotia holds promise and is fueled by products that directly align with consumer demand. This growth potential calls for continued efforts to refine methodologies, strengthen local food networks, and educate consumers. Challenges like seasonality and supply chain management deserve attention, while innovative solutions and collaborative partnerships will play pivotal roles in shaping a sustainable and thriving local food ecosystem.

## Figures and Tables

**Table 1:** Food outlets participants would use most and frequency of purchase.

Outlets	Never	Sometimes	Mostly	Always (Highest First)
Local supermarkets (e.g., Sobeys, Superstore)	0.74%	25.71%	56.61%	16.94%
Seasonally, my own backyard (I grow it)	40.56%	42.35%	7.73%	9.36%
Major supermarkets (e.g., Walmart)	19.91%	54.98%	18.42%	6.69%
Seasonally, community gardens (I grow it)	76.37%	17.98%	2.67%	2.97%
Local specialty stores (butcher shop, bakery, fruit and veg store)	21.10%	68.50%	8.47%	1.93%
Farmers’ market	24.67%	69.69%	4.61%	1.04%
Seasonally, roadside stands	26.45%	70.28%	2.23%	1.04%
General online stores (online grocery stores, Amazon, etc.)	71.62%	26.89%	0.59%	0.89%
Local general stores	40.27%	52.60%	6.24%	0.89%
Seasonally, friends or neighbours	42.79%	54.23%	2.38%	0.59%
Directly from farmers (box, farmgate, direct delivery)	69.99%	27.04%	2.67%	0.30%
Community-supported agriculture (CSA)	87.22%	10.55%	1.93%	0.30%
Online individual producers	84.70%	14.56%	0.59%	0.15%

**Table 2:** Money spent on food in total and on local food per month (in CAD).

Type of Food	Minimum	Maximum	Mean (CAD)	Std Deviation	Variance
Food in general (per month)	0.00	2000.00	681.12	404.81	163,868.54
Local food based on definitions (per month)	0.00	2000.00	317.76	355.12	126,110.94

Note: Both types exclude restaurants or take-out meals.

**Table 3:** Nova Scotia food expenditure data for 2019 from Statistics Canada in CAD and percentages.

Province: Nova Scotia	CAD	% Total Food
Total food purchased from store (without alcohol)	5001	100%
Processed products (bakery products)	645	13%
Cereal grains and cereal products	385	8%
Fruit, fruit preparations, and nuts	696	14%
Vegetables and vegetable preparations	792	16%
Dairy products and eggs (two categories)	969	19%
Meat	1345	27%
Fish and seafood	170	3%

Note: Some category names have been changed to match our local food categories.

**Table 4:** Results with Table 5 data for each food category in percentages.

Category	Mean	Min	Q1	Median	Q3	Maximum	SDev	Variance
dairy	48.39766	0	12.752803	39.95249	87.61029	100	36.77168	1352.1563
fruit	32.71176	0	9.000000	25.00000	50.00000	100	29.54816	873.0936
grain	21.50197	0	0.000000	4.00000	25.00000	100	33.88561	1148.2347
honey	40.05709	0	1.000000	9.00000	100.00000	100	45.18900	2042.0460
maple	36.74359	0	1.000000	7.00000	100.00000	100	43.91864	1928.8472
meat	34.16921	0	7.492495	21.67944	55.22978	100	32.76883	1073.7961
proc	22.45669	0	1.000000	7.00000	30.00000	100	31.72391	1006.4064
seafood	43.81299	0	5.000000	32.50000	90.25000	100	39.89036	1591.2411
veg	37.05894	0	10.000000	29.00000	59.00000	100	30.06306	903.7879
Total With SM	34.38238	0	13.81417	29.2609	51.61	98.42	23.82762	567.7555
Total w/o SM	31.17359	0	11.45679	26.37062	44.78417	98.17284	23.39106	547.1416

**Table 5:** Summary of averages from our three methods, with Method 2 being the most accurate.

Method	Estimate in % Per Month Spent on Local Food
Method 1: Total dollars spent on local food/Total dollars spent on food	46.7%
Method 2: Self-reported for food categories (without supply-managed categories)	31.2%
Method 3: Self-reported estimate in %	34.8%
Average of the three methods	37.6%

According to our sample size and the population of Nova Scotia, the margin of error for this study was 4.4% in 19 cases out of 20 (95% confidence interval). With this in mind, the floor and ceiling would be 33.16% and 41.96% for the average of the three methods.

## Data Availability

Data for this study are unavailable.

## Appendix A. Final Nova Scotia Local Food Definitions, December 2022

Nova Scotia Beef	Must be born and raised in Nova Scotia. Processing and packaging must be performed in Nova Scotia or in a facility approved by Nova Scotia.
Nova Scotia Lamb	Must be born and raised in Nova Scotia. Processing and packaging must be performed in Nova Scotia or in a facility approved by Nova Scotia.
Nova Scotia Pork	Must be born and raised in Nova Scotia. Processing and packaging must be performed in Nova Scotia or in a facility approved by Nova Scotia.
Nova Scotia Chicken	Must be hatched from eggs laid in Nova Scotia or from newly hatched chicks, which may be sourced from within Canada. These chickens must then be raised, processed, and packaged in Nova Scotia or in a facility approved by Nova Scotia.
Nova Scotia Turkey	Newly hatched turkey poults can be sourced within Canada. They must then be raised, processed, and packaged in Nova Scotia or in a facility approved by Nova Scotia.
Nova Scotia Fish and Seafood	Fish and seafood caught in Nova Scotia waters (defined by commercial fishing zones) or on sea farms in Nova Scotia. Processed and packaged in Nova Scotia.
Nova Scotia Cheese and Nova Scotia Dairy Products (yogurt, sour cream, etc.—excludes milk)	Cheese or milk products made with a minimum of 90% milk from Nova Scotia dairy farms. The rest of the milk must come from within Canada. All other ingredients must be sourced in Nova Scotia. Processing and packaging must be performed in Nova Scotia.
Nova Scotia Milk	A minimum of 90% of the milk must come from Nova Scotia dairy farms. The rest can come from within Canada. Processing and packaging must be performed in Nova Scotia.
Nova Scotia Eggs	Must be laid on egg farms in Nova Scotia. Processing and packaging must be performed in Nova Scotia.
Nova Scotia Fruits	Must be grown in Nova Scotia. Processing and packaging must be performed in Nova Scotia.
Nova Scotia Vegetables	Must be grown in Nova Scotia. Processing and packaging must be performed in Nova Scotia.
Nova Scotia Grains and Oil Seeds	A minimum of 80% of the product must be grown in Nova Scotia. The rest can come from within Canada. Processing and packaging must be performed in Nova Scotia or elsewhere in Canada. For wheat in particular, it must also be 100% milled in Nova Scotia.
Nova Scotia Honey	One hundred percent of the product must be produced, extracted, processed, and packaged in Nova Scotia.
Nova Scotia Maple Syrup	One hundred percent of the product must be collected, processed, and packaged in Nova Scotia.
Nova Scotia Processed Food Products	A minimum of 85% of the ingredients must come from Nova Scotia, and all processing and packaging must be performed in Nova Scotia.
